# Association between ultra-processed foods intake and frailty risk in community-dwelling older adults

**DOI:** 10.1186/s41043-025-01209-2

**Published:** 2025-12-25

**Authors:** Jamal Hallajzadeh, Parasto Yousefi Tanha, Arian Azadnia, Alexei Wong, Sajjad Moradi

**Affiliations:** 1 Research Center for Evidence-Based Health Management, Maragheh University of Medical Sciences, Maragheh, Iran; 2 Mahabad Faculty of Medical Sciences, Urmia University of Medical Sciences, Urmia, Iran; 3 Department of Community Nutrition, School of Nutritional Sciences and Dietetics, Tehran University of Medical Sciences (TUMS), Tehran, Iran; 4 Department of Epidemiology & Biostatistics, school of Medicine, Kurdistan University of Medical Sciences, Sanandaj, Iran; 5 Department of Health and Human Performance, Marymount University, Arlington, VA USA; 6 Department of Nutrition and Food Sciences, Maragheh University of Medical Sciences, Maragheh, Iran

**Keywords:** Ultra-processed foods, Frailty, Older adults, Elderly

## Abstract

**Aim:**

A community-based cross-sectional study was conducted to explore the association between Ultra-Processed Foods (UPFs) intake and the risk of frailty among community-dwelling older adults.

**Methods:**

The current study was conducted on 368 community-dwelling older adults (with a mean age of 67.11 ± 6.21 years, of whom 55.2% were women) at health centers of Maragheh city in Iran. Body composition was measured by a body composition analyzer and physical activity by the short-form physical activity questionnaire. The UPFs intake were determined using NOVA classification, based on a self-administered 147-item semi-quantitative FFQ. Blood samples were derived for the evaluation of blood parameters. Raw and adjusted logistic regression models were used to examine the relationship between UPFs intake tertiles and the risk of frailty.

**Results:**

Outcomes showed that the overall prevalence of frailty was 96 (26.1%). Results from the multivariable adjusted logistic regression model indicated that higher UPFs intake was significantly associated with higher odds of frailty (OR = 2.15, 95% CI: 1.13–4.09, *P* = 0.019). Subgroup analysis also indicated that among men, higher UPF intake was significantly related to higher odds of frailty (OR = 3.55, 95% CI: 1.20–10.51, *P* = 0.022), but not for women (*P* > 0.05). Additionally, the results revealed that higher UPF intake was significantly associated with the risk of exhaustion (OR = 3.97, 95% CI: 1.89–8.34, *P* < 0.001), especially among men (OR = 9.89, 95% CI: 3.10–31.60, *P* < 0.001), unlike women (*P* > 0.05). However, there were no significant associations between UPFs intake and other components of frailty, including the risk of weight loss, slowness, dominant hand grip strength and low physical activity.

**Conclusions:**

The results highlighted that higher UPFs intake was significantly associated with the risk of frailty and exhaustion among community-dwelling older adults, especially for men. Future large-scale prospective and interventional studies are warranted to validate these associations and elucidate underlying biological mechanisms.

**Supplementary Information:**

The online version contains supplementary material available at 10.1186/s41043-025-01209-2.

## Introduction

Frailty is a major health concern for older adults, characterized by reduced physiological reserve and a heightened susceptibility to adverse health outcomes [1, 2]. It is identified by symptoms such as fatigue, limited physical activity, muscle weakness, unintended weight loss and sluggish walking pace [3]. Frailty has been linked to higher rates of chronic illness, hospitalization and mortality; therefore, understanding its underlying causes is crucial as populations age worldwide. This need for insight underscores the significance of investigating factors that contribute to frailty, as undertaken in the present study.

Diet is one of the most critical factors affecting frailty [4]. According to recent studies, dietary habits significantly influence older persons’ health outcomes [5]. In particular, consumption of ultra-processed foods (UPFs) has drawn attention due to its potentially deleterious effects on physical and mental well-being [6–8]. UPFs are industrially produced goods lacking the vital nutrients in whole foods and frequently contain artificial substances, preservatives and additives [9]. These foods are typically high in added sugars, unhealthy fats and sodium, which can contribute to obesity and metabolic diseases [8, 10, 11]. Indeed, high intake of UPFs has been associated in several studies with an increased risk of chronic illnesses such as obesity, diabetes and cardiovascular disorders [8, 10–13]. However, little research has been done on the connection between UPFs and frailty [14, 15]. Investigating how UPFs consumption may impact frailty is crucial, especially since many older adults rely on convenience foods due to decreased mobility, financial constraints or limited access to fresh produce [16–18].

In addition to dietary intake, other factors such as physical activity levels, socioeconomic status and underlying health conditions also play a significant role in frailty development [19–21]. Regular physical activity increases muscle strength, improves balance, and supports overall health [22, 23], which may help counteract the effects of a diet high in UPFs. Moreover, socioeconomic status can influence dietary choices and access to healthy food options [24, 25], further complicating the relationship between diet and frailty. Recent evidence, including observational studies and comprehensive reviews, further motivates examining the relationship between UPFs and functional aging (frailty) in older adults [12, 26–28].

The novelty of this study lies in its focus on Iranian older adults, where unique dietary patterns, cultural practices, and the rapid growth of industrial food products may influence frailty risk differently than in Western populations. To address this gap in knowledge, the present study examines the association between UPFs consumption and frailty risk among community-dwelling older adults. We hypothesize that greater UPFs consumption is associated with greater frailty risk, even after controlling for potential confounders such as physical activity and socioeconomic status.

## Methods and materials

### Study population

The study was conducted between October 2024 to February 2025 in five primary health-care centers in Maragheh, Azarbaijan Sharghi, Iran. Sampling was performed using convenience sampling. The protocol of this study was approved by the local research ethics committees of Maragheh University of Medical Sciences (Ethical number: IR.MARAGHEHPHC.REC.1403.031, approval date: 07/14/2024). The inclusion criteria were as follows: community-dwelling older adults (aged 60 years old and above), volunteers who were willing to enroll in the project, those who were able to communicate were selected as study participants. Exclusion criteria also were: those subjects who were dissatisfied with continuing to study, individuals with inability to move independently and dependent on a walker, wheelchair or bed, participants with an artificial limb or prosthesis, those with severe muscle-related and debilitating effects such as congestive heart failure (CHF), chronic obstructive pulmonary disorder (COPD), chronic renal failure (CRF), cirrhosis and liver failure (based on personal report or medical record) and inflammatory and joint diseases including arthritis, rheumatoid arthritis, bursitis, inflammatory bowel disease, Guillain-Barré syndrome and cancer (based on personal report or medical record).The older adults read and signed the informed consent before taking part in study. Trained researchers performed interviews with subjects to decrease interviewer bias.

### Demographics

Demographic parameters, including age, sex, education level, smoking status and marital status, were gathered via a general questionnaire.

## Complete body composition analysis

Body composition was evaluated by well-trained researchers utilizing the TANITA BC-601 (Japan) under standardized fasting conditions in the morning, according to manufacturer guidelines and peer-reviewed validation studies [29, 30]. The device calculates body fat percentage, fat mass, fat-free mass and predicts muscle mass based on data obtained via Bioelectrical Impedance Analysis, a method validated against the gold standards of Dual-Energy X-Ray Absorptiometry and Underwater Weighing [29]. All BIA measurements were performed in the morning after an overnight fast of at least 8 h, with participants having voided their bladder immediately prior to the measurement, wearing light clothing and no shoes, and having removed metal accessories. Participants were instructed to avoid moderate-to-vigorous exercise for 12 h and to refrain from alcohol in the preceding 24 h; these procedures follow manufacturer recommendations and established BIA guidance. The TANITA device provides estimates of body fat percentage, fat mass and fat-free mass using foot-to-foot impedance; the method was applied consistently and the same calibrated device was used throughout the study.

### Anthropometric assessment

Weight (with an accuracy of 100 g) was measured with minimal clothing and without shoes using a Seca digital scale (Seca 803, Germany). Height (with an accuracy of 0.5 cm) was quantified utilizing a Seca height gauge (Seca 206, Germany) with the participant standing against a wall and without shoes, ensuring that the shoulders were in a neutral position. Body Mass Index (BMI) was calculated as weight (kg) divided by height squared (m²). Waist circumference was measured with an inelastic tape measure (accuracy: 0.5 cm), at the midpoint between the last rib and the iliac crest, with the participant in a standing position at the end of a natural exhalation. Hip circumference was determined by means of a tape measure at the widest point of the hips using standard procedures.

### Ultra-processed foods assessments

Dietary intake was assessed with the use of a valid and reliable 147-item semi-quantitative food frequency questionnaire (FFQ) [31], which evaluated participants’ usual food intake over the preceding 12 months. The consumption frequency of each food item on a daily, weekly or monthly basis was converted to daily intakes; portion sizes were then converted to grams, using household measures. The portion sizes were estimated using standard Iranian household measures, converted to grams using TLGS protocols and the Iranian food composition tables. The daily intake of UPFs was determined according to the NOVA food group classification [32, 33], by summing the daily intake of 31 food and beverage items classified as UPFs, and expressing this as intake in grams per day. To estimate the contribution of UPFs to total energy intake, the daily intake of each UPF was multiplied by its energy content. The total caloric intake from UPFs was then calculated by summing the calories from all UPF items. The energy from UPFs was calculated by multiplying grams/day of each UPF item by its energy density (kcal/g) and summing, then dividing by total daily energy intake to compute percentage of energy from UPFs. The full list of 31 items is provided in Supplementary Table 1. Previous studies have validated this approach for UPF assessment [34–37].

### Physical activity

The physical activity was evaluated by the short-form International Physical Activity Questionnaire (IPAQ), which is an instrument designed primarily for population-level physical activity surveillance in adults [38]. The validity and reliability of this questionnaire have been well-established [39]. This form consists of seven questions that measure physical activity in four levels: vigorous, moderate, walking and inactivity.

### Sleep quantity

Sleep duration was assessed employing the Pittsburgh Sleep Quality Index (PSQI) questionnaire [40], a widely used tool for evaluating sleep quality and duration.

### Socioeconomic status

Socioeconomic status (SES) was assessed via the socioeconomic status short-form (SES-SQ) questionnaire, a valid and reliable tool [41]. This questionnaire comprises six items related to housing status, employment status, vehicle ownership, use of electronic devices and travel status. The SES-SQ has a total score of 17 points. A score higher than 8.5 indicates favorable socioeconomic status, while a score lower than 8.5 indicates unfavorable socioeconomic status.

### Biochemical and inflammatory markers levels assay

All blood samples were collected between 8:00 and 10:00am following an overnight fasting. Serum samples were centrifuged and aliquoted, then stored at −80 °C prior to analysis. Serum IL-6 (LDN, Germany) and h-CRP (Aptec, Belgium) concentrations were assessed using the enzyme-linked immunosorbent assay (ELISA) following the manufacturer’s protocols. Routine biochemical parameters included fasting blood glucose (FBG), lipid profile [total cholesterol (TC), triglycerides (TG), high-density lipoprotein cholesterol (HDL-C) and low-density lipoprotein cholesterol (LDL-C)] as well as liver enzymes [alanine aminotransferase (ALT), aspartate aminotransferase (AST) and alkaline phosphatase (ALP)]. Analyses were carried out using commercial kits (Dialab, Austria) on an automated analyzer (Mindray BS-430, China). LDL-C was calculated using the Friedewald equation (LDL-C = TC − HDL-C − TG/5) for samples with TG concentrations below 400 mg/dL.

### Frailty assessment

Frailty in older adults was evaluated applying Fried’s criteria [42]. This standardized assessment includes five components, yielding a cumulative score ranging from 0 to 5. In alignment with the study objective to identify individuals classified as frail, participants meeting at least three of the following criteria were considered frail. The components are described as follows:

1- Exhaustion (Self-Reported Fatigue): This criterion was assessed via self-report. Participants were queried regarding their subjective feelings of fatigue. Individuals reporting persistent tiredness, feelings of helplessness, or difficulty moving and working for the majority of the week (example: 3 to 4 days) received a score for this criterion.

2- Muscle weakness: Muscular strength was assessed bilaterally using a handgrip dynamometer (Constant 709-E, China). Each hand was tested twice for 30 s. The obtained score (kg) was compared against standardized reference values stratified by sex and body mass index. Values below the threshold indicate weakness and result in a positive score for this criterion. The lowest handgrip value below the cutoff was sufficient to classify weakness.

3- Low walking speed: Gait speed was measured over a 4.5-meter course, performed twice. The fastest time was recorded and compared to normative reference values based on the individual’s height and sex. Participants whose gait speed exceeds the threshold (slower walking time) were assigned a score for this criterion.

4- Very low physical activity: The caloric expenditure for physical activity is less than 383 in men and less than 270 kcal in women.

5- Unintentional weight loss: This criterion was defined as an involuntary weight loss exceeding 4.5 kg over the past year.

### Sample size

To estimate the prevalence of frailty syndrome in older adults, the sample size was calculated using the following formula for prevalence studies:

$$n = \frac{Z^{2} \cdot P \cdot (1 - P)}{d^{2}}$$ 

 Where: n = required sample size

 = Z-value corresponding to the desired confidence level (for a 95% confidence interval, Z=1.96)

 P = expected prevalence (based on the study Boura et al. 2021 [43], P=0.35 or 35%, with 1−P=0.65)

d = margin of error (set at 0.05 or 5%).

Using these parameters, the initial required sample size was approximately 350 participants. To account for a potential dropout rate of 5%, the sample size was recalculated using the following formula:

$$n_\mathrm{(adjusted)}=\frac {n}{(1-dropout\, rate)}$$ 

Where:

n = initial sample size (350).

Dropout rate = 0.05 (5%).

Rounding to the nearest whole number, the final sample size was determined to be 368 participants.

### Statistical analysis

Data analysis was performed using SPSS version 24 software. Descriptive statistics, including mean, standard deviation, frequency, relative frequency and related graphs were used to summarize the data. The type of analysis performed was dependent on the nature of the variables. The chi-square test or Fisher’s exact test was applied to examine the relationship between qualitative variables. For the relationship between quantitative variables and binary outcome variables, independent t-tests and one-way ANOVA (or their nonparametric equivalents) were employed. Logistic regression models were the primary method, and we additionally performed Poisson regression with robust variance to estimate risk ratios. Fat percentage and fat mass were analyzed in separate models to avoid collinearity. Socioeconomic status was included in the adjusted regression models, while physical activity (a frailty component) was only adjusted in sensitivity analyses, not in the primary models to avoid over-control. Missing data were < 5% and handled by complete-case analysis.

## Results

### Participant’s characteristics

The study comprised 368 participants with a mean age of 67.11 ± 6.21 years, of whom 55.2% were women. Participants were stratified into tertiles (T1, T2, T3) based on their UPFs intake score, with each tertile including approximately 122–123 participants. Table 1 summarizes the baseline characteristics across these tertiles. Significant differences were observed across tertiles for several variables. Age varied significantly (*P* = 0.019), with T1 exhibiting the highest mean age (68.30 ± 6.27 years) and T3 the lowest (66.11 ± 6.06 years). Total calorie intake was significantly higher in T3 (2945.21 ± 660.97 kcal/day) compared to T1 (2787.69 ± 563.30 kcal/day) and T2 (2703.26 ± 484.38 kcal/day; *P* = 0.004). Body fat percentage (FAT) and fat mass were significantly higher in T3 (32.19 ± 8.46% and 23.63 ± 8.06 kg, respectively) compared to T1 (29.52 ± 7.77% and 21.10 ± 7.17 kg; *P* = 0.029 and *P* = 0.013, respectively). High-sensitivity C-reactive protein (h-CRP) levels differed significantly (*P* = 0.014), with T2 showing the highest levels (11.15 ± 6.87 mg/L) compared to T1 (8.80 ± 5.94 mg/L) and T3 (9.75 ± 5.98 mg/L). Cholesterol levels were significantly higher in T3 (190.44 ± 46.14 mg/dL) compared to T1 (172.95 ± 42.09 mg/dL; *P* = 0.008). Exhaustion prevalence was significantly higher in T3 (30.1%) compared to T1 (9.8%; *P* < 0.001). Frailty prevalence also differed significantly (*P* = 0.039), with T3 showing the highest prevalence (31.7%) compared to T1 (18%) and T2 (28.5%). Missing data analysis revealed the following missingness patterns: h-CRP (1.2%), IL-6 (1.1%), biochemical markers (1.4%), and dietary variables (1.6%). Complete-case analysis was our primary approach due to low overall missingness(< 5%).

**Table 1:** Participant’s characteristics by UPFs intake score quartiles

Variables	All participants (*N* = 268)	T1 (*N* = 123)	T2 (*N* = 123)	T3 (*N* = 122)	*P*-value
Age (years)	67.11 ± 6.21^1^	68.30 ± 6.27	66.91 ± 6.19	66.11 ± 6.06	0.019
Weight (kg)	72.38 ± 11.90	70.58 ± 10.80	73.67 ± 12.40	72.89 ± 12.31	0.107
BMI (kg/m2)	27.14 ± 4.51	26.34 ± 4.11	27.56 ± 4.74	27.54 ± 4.59	0.053
Sex (F%)					
Male	165(44.8)^2^	63(51.2)	55(44.7)	47(38.5)	0.136
Female	203(55.2)	60(48.8)	68(55.3)	75(61.5)	
Total calorie intake (Kcal/d)	2811.69 ± 581.01	2787.69 ± 563.30	2703.26 ± 484.38	2945.21 ± 660.97	0.004
Waist circumference (cm)	97.16 ± 10.44	96.08 ± 10.08	97.60 ± 11.107	97.79 ± 10.09	0.375
Hip circumference (cm)	107.38 ± 9.56	106.80 ± 8.84	107.26 ± 10.18	108.09 ± 9.64	0.567
Fat (%)	31.16 ± 8.50	29.52 ± 7.77	31.79 ± 9.03	32.19 ± 8.46	0.029
Fat free mass (kg)	49.41 ± 8.19	49.48 ± 7.90	49.78 ± 8.27	48.98 ± 8.45	0.745
Visceral Fat (%)	11.62 ± 3.58	11.61 ± 3.46	12.08 ± 3.99	11.23 ± 3.22	0.243
Fat mass (kg)	22.87 ± 8.22	21.10 ± 7.17	23.89 ± 9.09	23.63 ± 8.06	0.013
Physical activity levels (MET)	808.65 ± 673.37	803.13 ± 696.37	736.14 ± 681.16	887.33 ± 637.88	0.213
AST (U/L)	20.97 ± 15.30	22.89 ± 24.40	21.08 ± 8.37	19 ± 5.74	0.140
ALT (U/L)	23.67 ± 14.03	24.89 ± 21.45	23.96 ± 8.73	22.14 ± 7.11	0.297
IL6 (pg/mL)	15.33 ± 12.21	14.67 ± 14.19	16.67 ± 10.38	14.64 ± 11.75	0.329
h-CRP (mg/L)	9.90 ± 6.34	8.80 ± 5.94	11.15 ± 6.87	9.75 ± 5.98	0.014
Alkaline phosphatase (IU/L)	184.96 ± 49.22	181.14 ± 47.34	180.42 ± 50.55	193.44 ± 49.01	0.068
FBS (mg/dl)	112.31 ± 42.53	117.02 ± 52.24	108.86 ± 28.22	111.09 ± 43.63	0.301
HDL-C (mg/dl)	48.37 ± 9.86	47.50 ± 9.41	48.26 ± 10.50	49.38 ± 9.62	0.329
LDL-C (mg/dl)	102.49 ± 38.85	97.80 ± 38.76	100.93 ± 38.08	108.85 ± 39.20	0.074
Cholesterol (mg/dl)	180.75 ± 44.96	172.95 ± 42.09	178.96 ± 45.15	190.44 ± 46.14	0.008
Triglycerides (mg/dl)	150.39 ± 64.95	142.04 ± 55.9	148.08 ± 63.41	161.24 ± 73.47	0.062
Socioeconomic status score	7.37 ± 2.34	7.33 ± 2.24	7.26 ± 2.54	7.52 ± 2.24	0.676
Education					
Illiterate	39(10.6)	15(12.2)	10(8.1)	14(11.5)	0.953
Under Diploma	125(34)	39(31.7)	44(35.8)	42(34.4)	
Diploma	83(22.6)	27(22)	29(23.6)	27(22.1)	
University degree	121(32.9)	42(34.1)	40(32.5)	39(32)	
Marital status					
Married	324(88)	110(89.4)	107(87)	107(87.7)	0.83
Single	44(12)	13(10.6)	16(13)	15(12.3)	
Health status					
Healthy participants	96 (26.2)	26(7.1)	35(9.5)	35(9.5)	0.247
Diabetes	67(18.2)	26(7.1)	26(7.1)	15(4.1)	
Hypertension	117(31.8)	38(10.3)	34(9.2)	45(12.2)	
Cardiovascular	18(4.9)	10(2.7)	5(1.4)	3(0.8)	
Hypothyroidism	5(1.4)	-	2(0.5)	3(0.8)	
Multimorbidity (presence 2 or more long-term health conditions)	34(9.2)	13(3.5)	11(3)	10(2.7)	
Others	31(8.4)	9(2.4)	10(2.7)	12(3.3)	
Others					
Sleep_duration					
Less than 5 H	30(8.2)	7(5.7)	13(10.6)	10(8.2)	0.344
5–6 H	75(20.4)	22(17.9)	24(19.5)	29(23.8)	
6–7 H	107(29.1)	32(26)	36(29.3)	39(32)	
More than 7 H	156(42.4)	62(50.4)	50(40.7)	44(36.1)	
Smoking					
NO	327(88.9)	108(87.8)	113(91.9)	106(86.9)	0.418
Yes	41(11.1)	15(12.2)	10(8.1)	16(13.1)	
Weight loss					
No	288(78.3)	98(80.3)	98(79.7)	92(74.8)	0.519
Yes	80(21.7)	24(19.7)	25(20.3)	31(25.2)	
Slowness					
No	296(80.4)	99(80.5)	98(79.7)	99(81.1)	0.959
Yes	72(19.6)	24(19.5)	25(20.3)	23(18.9)	
Low muscle strength					
No	141(38.3)	52(42.3)	47(38.2)	42(34.4)	0.450
Yes	227(61.7)	71(57.7)	76(61.8)	80(65.6)	
Low physical activity					
No	275(74.5)	97(78.9)	85(69.1)	92(75.4)	0.205
Yes	94(25.5)	26(21.1)	38(30.9)	30(24.6)	
Exhaustion					
No	302(82.1)	110(90.2)	106(86.2)	86(69.9)	< 0.001
Yes	66(17.9)	12(9.8)	17(13.8)	37(30.1)	
Frailty					
No	272(73.9)	100(82)	88(71.5)	84(68.3)	0.039
Yes	96(26.1)	23(18)	35(28.5)	39(31.7)	

MET: Metabolic equivalent of task, BMI: Body mass index, T: Tertile, AST: Aspartate aminotransferase, ALT: Alanine aminotransferase, IL-6:Interleukin‐6, h-CRP: High-sensitivity C-reactive protein, FBS: Fasting blood sugar, HDL-C: high-density lipoprotein cholesterol, LDL-C:

1 Variables are expressed as mean ± SD

2 Variables are expressed as n (%)

(Chi-square/Fisher’s exact test for categorical variables, ANOVA for continuous variables)

No significant differences were found for body mass index (BMI), weight, waist or hip circumference, fat-free mass (FFM), visceral fat, physical activity levels (MET), aspartate aminotransferase (AST), alanine aminotransferase (ALT), interleukin-6 (IL-6), fasting blood sugar (FBS), high-density lipoprotein cholesterol (HDL-C), low-density lipoprotein cholesterol (LDL-C), triglycerides, socioeconomic status (SES), education, marital status, sleep duration, subjective sleep quality, smoking status, weight loss, slowness, dominant hand grip strength, or low physical activity (*P* > 0.05).

Table 2 presents the odds ratios (ORs) and 95% confidence intervals (CIs) for the association between UPFs intake and frailty-related outcomes, using T1 as the reference group. Both crude and adjusted logistic regression models were employed, with adjustments for sex, age, BMI, FAT, fat mass, AST, h-CRP, alkaline phosphatase, LDL-C, cholesterol, and triglycerides.

**Table 2:** Odds ratios (ORs) and 95% confidence intervals (CIs) for the association between UPFs intake and risk of Frailty. Results from logistic regression multivariable model (T1 as reference group)

Frailty outcomes	Crud model OR(%95 CI), *P*-value						Adjusted model OR(%95 CI), *P*-value					
	**T1**	**T2**	**P-value**	**T3**	**P-value**	P _Trend_	**T1**	**T2**	**P-value**	**T3**	P-value	P _Trend_
**Weight loss**												
All (*N* = 368)	Ref.	1.04(0.57–1.94)^1^	0.898	1.31(0.71–2.41)	0.374	0.613	Ref.	1.25(0.64–2.43)	0.499	1.62(0.83–3.16)	0.156	0.594
Men (*N* = 165)	Ref.	0.34(0.11–1.02)	0.055	1.14(0.47–2.76)	0.767	0.560	Ref.	0.29(0.88–1.00.88.00)	0.050	1.72(0.63–4.68)	0.286	0.174
Women (*N* = 203)	Ref.	2.08(0.88–4.90)	0.093	1.57(0.66–3.73)	0.299	0.366	Ref.	3.17(1.21–8.28)	0.018	2.06(0.76–5.58)	0.151	0.061
**Slowness**												
All (*N* = 368)	Ref.	1.05(0.56–1.96)	0.873	0.95(0.50–1.81)	0.896	0.897	Ref.	1.01(0.52–1.96)	0.975	0.82(0.41–1.63)	0.574	0.800
Men (*N* = 165)	Ref.	1.85(0.72–4.75)	0.197	0.71(0.22–2.29)	0.571	0.721	Ref.	1.52(0.52–4.44)	0.436	0.59(0.16–2.14)	0.425	0.308
Women (*N* = 203)	Ref.	0.64(0.27–1.51)	0.311	0.94(0.43–2.08)	0.893	0.964	Ref.	0.66(0.27–1.60)	0.359	0.83(0.35–1.96)	0.831	0.654
**Low muscle strength**												
All (*N* = 368)	Ref.	1.18(0.71–1.97)	0.516	1.39(0.83–2.34)	0.207	0.141	Ref.	1.34(0.76–2.35)	0.298	1.55(0.87–2.75)	0.131	0.167
Men (*N* = 165)	Ref.	2.05(0.97–4.29)	0.057	1.33(0.62–2.84)	0.458	0.922	Ref.	2.96(1.23–7.12)	0.015	1.53(0.63–3.69)	0.342	0.657
Women (*N* = 203)	Ref.	0.65(0.31–1.35)	0.252	1.17(0.55–2.5)	0.669	0.123	Ref.	0.77(0.34–1.69)	0.517	1.46(0.64–3.33)	0.367	0.028
**Low physical activity**												
All (*N* = 368)	Ref.	1.66(0.93–2.97)	0.083	1.21(0.66–2.21)	0.520	0.534	Ref.	1.50(0.81–2.74)	0.190	1.07(0.57–2.02)	0.821	0.358
Men (*N* = 165)	Ref.	2.03(0.88–4.63)	0.093	1.04(0.41–2.62)	0.935	0.814	Ref.	1.58(0.65–3.82)	0.305	0.88(0.32–2.37)	0.802	0.407
Women (*N* = 203)	Ref.	1.40(0.62–3.15)	0.414	1.31(0.59–2.92)	0.502	0.533	Ref.	1.36(0.57–3.25)	0.477	1.28(0.53–3.09)	0.569	0.763
**Exhaustion**												
All (*N* = 368)	Ref.	1.47(0.67–3.22)	0.336	3.94(1.94–8.01)	< 0.001	0.008	Ref.	1.38(0.62–3.09)	0.424	3.97(1.89–8.34)	< 0.001	0.081
Men (*N* = 165)	Ref.	1.14(0.31–4.16)	0.843	8.14(2.76–23.96)	< 0.001	0.002	Ref.	0.99(0.26–3.85)	0.996	9.89(3.10–31.60)	< 0.001	0.009
Women (*N* = 203)	Ref.	1.62(0.59–4.43)	0.345	1.58(0.59–4.27)	0.360	0.390	Ref.	1.78(0.59–5.31)	0.298	1.42(0.48–4.15)	0.518	0.582
**Frailty**												
All (*N* = 368)	Ref.	1.80(0.98–3.31)	0.055	2.11(1.16–3.83)	0.014	< 0.001	Ref.	1.84(0.97–3.48)	0.059	2.15 (1.13–4.09)	0.019	0.003
Men (*N* = 165)	Ref.	2.41(0.96–6.03)	0.059	2.94(1.16–7.44)	0.022	0.005	Ref.	2.41(0.86–7.10)	0.091	3.55(1.20–10.51.20.51)	0.022	0.020
Women (*N* = 203)	Ref.	1.40(0.62–3.15)	0.414	1.59(0.72–3.51)	0.242	0.050	Ref.	1.44(0.62–3.35)	0.390	1.47 (0.63–3.42)	0.364	0.152

### Weight loss

No significant associations were observed between UPFs intake and weight loss in the adjusted model for the overall population (T3: OR = 1.62, 95% CI: 0.83–3.16, *P* = 0.156, P_trend_= 0.594), men (T3: OR = 1.72, 95% CI: 0.63–4.68, *P* = 0.286, P_trend_= 0.174), or women (T3: OR = 2.06, 95% CI: 0.76–5.58, *P* = 0.151, P_trend_= 0.061). The crude model also showed no significant associations (*P* > 0.05).

### Slowness

No significant associations were found between UPFs intake and slowness in the overall population (T3: OR = 0.82, 95% CI: 0.41–1.63, *P* = 0.574, P_trend_= 0.800), men (T3: OR = 0.59, 95% CI: 0.16–2.14, *P* = 0.425, P_trend_= 0.360), or women (T3: OR = 0.83, 95% CI: 0.35–1.96, *P* = 0.831, P_trend_= 0.654) in the adjusted model. The crude model also showed no significant associations (*P* > 0.05).

### Low muscle strength

No significant associations were observed in the adjusted model for the overall population (T3: OR = 1.55, 95% CI: 0.87–2.75, *P* = 0.131, P_trend_= 0.167), men (T3: OR = 1.53, 95% CI: 0.63–3.69, *P* = 0.342, P_trend_= 0.658). For women, the adjusted model suggested a modest, non-significant increase in the odds of low muscle strength in the highest tertile (OR = 1.46, 95% CI: 0.64–3.33, *p* = 0.367). The p-trend value (*p* = 0.028) suggested a possible dose–response pattern, although the association did not reach statistical significance.

### Low physical activity

No significant associations were found between UPFs intake and low physical activity in the adjusted model for the overall population (T3: OR = 1.07, 95% CI: 0.57–2.02, *P* = 0.821, P_trend_= 0.358), men (T3: OR = 0.88, 95% CI: 0.32–2.37, *P* = 0.802, P_trend_= 0.407), or women (T3: OR = 1.28, 95% CI: 0.53–3.09, *P* = 0.569, P_trend_= 0.763).

### Exhaustion

Significant associations were observed for exhaustion in the overall population and men in the adjusted model. Participants in T3 had almost four times higher odds of exhaustion compared with T1 (OR = 3.97, 95% CI: 1.89–8.34, *p* < 0.001), although the overall p-trend was marginal (p-trend = 0.081). Men in T3 had significantly higher odds (OR = 9.89, 95% CI: 3.10–31.60, *P* < 0.001) compared to T1. The p-trend value (*p* = 0.009) suggested a possible dose–response pattern. In the crude model, these associations were also significant for the overall population (T3: OR = 3.94, 95% CI: 1.94–8.01, *P* < 0.001) and men (T3: OR = 8.14, 95% CI: 2.76–23.96, P = < 0.001). No significant associations were found for women in either model (T3: OR = 1.58, 95% CI: 0.59–4.27, *P* = 0.360 in adjusted model).

### Frailty

Significant associations were observed for frailty in the overall population in the adjusted model. In the adjusted model, individuals in the highest tertile of UPF intake had 2.15-fold higher odds of frailty compared to the lowest tertile (95% CI: 1.13–4.09, *p* = 0.019), with a significant dose–response trend (p-trend = 0.003). Men in T3 had significantly higher odds (OR = 3.55, 95% CI: 1.20–10.51, *P* = 0.022) but not for women. The p-trend value (*p* = 0.020) suggested a possible dose–response pattern. In the crude model, significant associations were found for the overall population (T3: OR = 2.11, 95% CI: 1.16–3.83, *P* = 0.014) and men (T3: OR = 2.94, 95% CI: 1.16–7.44, *P* = 0.022).

## Discussion

This community-based cross-sectional study provides robust evidence linking high consumption of ultra-processed foods (UPFs) to an elevated risk of frailty among older adults. Participants in the highest tertile of UPF intake (T3) were more than twice as likely to be classified as frail compared to those in the lowest tertile (T1), even after adjusting for a broad array of confounding variables. The observed significant p-trend values indicate a dose–response relationship between UPF intake and frailty/exhaustion, lending further support to the plausibility of these associations. These findings corroborate and extend prior research [14, 15, 44], reinforcing the emerging consensus that nutrient-poor, energy-dense UPFs (often laden with harmful additives) may accelerate biological aging processes and increase frailty vulnerability.

A novel observation in our study is the strong association between UPFs intake and the exhaustion component of frailty. Men in the highest UPFs intake tertile had nearly fivefold higher odds of experiencing exhaustion (extreme fatigue) than those in the lowest tertile (OR = 4.92, 95% CI: 1.62–14.88). Very limited studies have been published in this field so far. In cross-sectional study Hosseininasab et al. [45] reported that Iranian women those with the highest adherence to UPF intake, there was an 8.76 unit reduction in addressing feelings of energy and fatigue. This sex-specific disparity is consistent with literature indicating that older men may be more sensitive to metabolic and inflammatory dysregulation arising from poor dietary quality, possibly due to differences in body composition, hormonal profiles, and mitochondrial function [46]. Specifically, the reduced anti-inflammatory effect of testosterone, combined with the lack of estrogen’s antioxidant and anti-inflammatory effects in men [47–50], may place older men at greater physiological risk when consuming high-UPF diets. Moreover, chronic consumption of UPFs has been shown to impair mitochondrial bioenergetics [51], reduce ATP availability and increase oxidative stress [52]. Such changes are central to the development of fatigue and exhaustion [53, 54], which is often one of the earliest indicators of functional decline in older adults.

Beyond systemic inflammation and impaired energy metabolism, there are additional plausible biological pathways linking UPF consumption to frailty. First, chronic intake of UPFs can alter the gut microbiota (dysbiosis), reducing production of short-chain fatty acids and impairing gut barrier integrity, which in turn promotes systemic inflammation and metabolic dysregulation [55, 56]. Second, diet-driven systemic inflammation and microbial metabolites can contribute to neuroinflammation, which may affect central regulation of energy, motivation and fatigue pathways, thereby contributing to exhaustion and reduced physical performance [57, 58]. Third, the “loss of the food matrix” in many UPFs i.e., destruction of the natural cellular structure and food-associated nutrients/phytonutrients can reduce bioavailability of beneficial compounds and alter post-prandial metabolic responses, further impairing mitochondrial function and muscle energetics. These mechanistic hypotheses are consistent with experimental and translational work showing UPF-related effects on mitochondrial bioenergetics, oxidative stress and immune activation [59, 60].

Interestingly, other frailty components such as slowness, reduced grip strength and unintentional weight loss did not show statistically significant associations with UPF consumption in our analysis. This may indicate that energy metabolism–related domains (e.g., exhaustion) respond more acutely to dietary quality, whereas neuromuscular function and weight change might require longer exposure or be more strongly influenced by physical activity, chronic disease, or lifelong nutritional patterns. Alternatively, the cross-sectional design may limit detection of gradual declines in grip strength or gait speed that develop over years. These considerations underscore the need for longitudinal studies to determine how dietary exposures influence the progression of various frailty components over time.

Further supporting the biological plausibility of the UPF and frailty link, we found that higher UPFs consumption was associated with elevated high-sensitivity C-reactive protein (hs-CRP) and cholesterol (both markers of systemic inflammation and metabolic dysfunction). Adiposity appeared to mediate part of the association, as participants in the highest UPF tertile had greater fat mass, which is a recognized driver of insulin resistance, chronic inflammation and hormonal imbalance pathways that can impair muscle quality and promote sarcopenic obesity [28, 61, 62]. The pro-inflammatory milieu promoted by UPF-heavy diets (which are rich in refined carbohydrates, trans fats, and synthetic additives) [63, 64], is known to disrupt immune regulation, endothelial function and metabolic homeostasis [65]. Additionally, those in the highest UPFs tertile had greater fat mass, implicating adiposity as a potential mediator in the diet–frailty relationship. Excess adipose tissue is a key driver of insulin resistance, chronic inflammation and hormonal imbalances, which can impair muscle quality and contribute to sarcopenic obesity a condition recognized as a pathway to frailty in older adults [66].

Methodologically, the study is strengthened by using validated tools such as the FFQ and Fried’s frailty phenotype and by its comprehensive adjustment for confounding variables. The stratified analysis by sex adds depth to the findings, suggesting the crucial need for gender-sensitive dietary interventions. However, several limitations must be acknowledged. Chief among these is the cross-sectional design, which precludes causal inference and leaves open the possibility of reverse causation. For example, it is plausible that frail individuals might consume more UPFs due to convenience (stemming from reduced mobility or energy) or due to limited access to fresh foods. Furthermore, although we used the NOVA classification to quantify UPF intake, this system has been criticized for oversimplifying food quality by not distinguishing nutrient-fortified foods or the wide variability within processed foods. In addition, dietary data were collected using an FFQ, which is subject to recall bias and measurement error. The generalizability of our findings may also be limited, as the study population consisted of individuals from a single urban area and with a relatively young older age profile (mean age 67 years), which may not reflect dietary patterns or frailty outcomes in more diverse or much older populations. Future research would benefit from more granular dietary assessment tools to capture the complexity of modern food environments and to verify whether the observed associations hold when using alternative dietary quality metrics.

From a public health perspective, the findings of this study carry significant implications. As global aging accelerates [67] and UPFs become increasingly prevalent in diets worldwide [68], identifying modifiable dietary risk factors for frailty becomes an urgent priority. Our results suggest that targeted nutritional strategies such as limiting the availability of UPFs in settings frequented by older adults, subsidizing healthier minimally processed foods and educating the public about the risks of high-UPF diets could help prevent or delay frailty onset. These efforts may be particularly impactful among men, who appear more susceptible to diet-related fatigue and functional decline.

The Iranian dietary context differs substantially from Western populations studied previously [69–72]. Traditional Persian cuisine emphasizes rice, legumes, and minimally processed ingredients, yet rapid urbanization has increased UPF availability [72, 73]. Our finding of 26.1% frailty prevalence is higher than reported in Mediterranean populations (15–20%) but similar to industrialized Asian countries experiencing nutrition transition [74]. Cultural-related factors such as excessive sodium consumption and higher prevalence nutritional zinc-deficiency in men than women [75, 76] may intensify UPF effects through stimulate dehydration [77, 78] or impaired physical function [79, 80] respectively, potentially explaining why our associations were primarily observed in men.

While our findings support reducing UPF consumption, practical implementation faces significant challenges. Industry resistance to reformulation, particularly in low-income settings where UPFs provide affordable calories, requires policy-level interventions including taxation of ultra-processed products and subsidization of whole foods. For vulnerable subgroups such as older adults living alone, meal delivery programs featuring minimally processed foods may be more effective than general dietary education.

Rural older adults face unique barriers including limited fresh food access and transportation difficulties [69]. Community-based interventions such as mobile farmers’ markets and cooking skill programs adapted for physical limitations may address these challenges. Gender-specific approaches are warranted, with particular attention to male-targeted nutrition education given their higher susceptibility to UPF-related exhaustion.

Methodologically, our study has several strengths and limitations that we now state more objectively. Strengths include a community-based sample, use of validated instruments (FFQ, Fried’s frailty phenotype), and adjustment for multiple potential confounders. However, the cross-sectional design precludes causal inference and raises the possibility of reverse causation (for example, frail individuals may select more UPFs because of convenience). Measurement error is possible because dietary intake was assessed with an FFQ; although the NOVA classification is a useful tool to quantify UPF exposure, it can oversimplify heterogeneity within processed foods (e.g., fortified vs. non-fortified items). We did not include validated recovery biomarkers (such as urinary sucrose or plasma industrial trans-fatty acids) to quantify UPF exposure, which may lead to misclassification; future studies should consider such biomarkers.

We also acknowledge several unmeasured or incompletely measured confounders that could influence our results, and have added these explicitly to the limitations. Notably, polypharmacy, overall chronic disease burden (e.g., a comorbidity index), and measures of social support or household food access were not accounted for and could confound or modify the observed associations. Although physical activity and socioeconomic status were adjusted for, residual confounding by health status, mobility limitations, or medication use remains possible. Moreover, statistical power for sex-stratified analyses was limited; therefore, sex differences should be interpreted as exploratory.

Regarding analytic scope, we did not perform a comprehensive battery of sensitivity/subgroup analyses or formal mediation/moderation tests in the present manuscript. These analyses (for example: stratification by comorbidity status, exclusion of participants with severe chronic disease, adjustment for classes of medications, interaction tests by sex and age, and causal mediation analyses with hs-CRP or fat mass as mediators) are important next steps and are recommended for future work.

From a public health perspective, the findings carry important implications but require cautious translation into policy. Practical implementation of UPF-reduction strategies faces real challenges including industry resistance to reformulation, affordability of minimally processed foods for low-income groups, established eating habits, and limited access to fresh foods in some areas. Policy levers that could be considered include fiscal measures (taxes on highly processed products), subsidies or vouchers for whole/minimally processed foods, front-of-pack labeling, restrictions on targeted marketing to vulnerable groups, and support for community programs that increase access to fresh food. However, the effectiveness and equity implications of each policy should be evaluated locally before wide implementation.

Concrete, targeted interventions for vulnerable subgroups are likely to improve applicability. For older adults living alone or with mobility limitations, subsidized meal-delivery programs emphasizing minimally processed, nutrient-dense meals; community kitchens; or volunteer-based meal sharing could reduce reliance on UPFs. For rural older adults, interventions such as mobile farmers’ markets, local produce cooperatives, and transport support may be effective. Gender-sensitive approaches — for example, male-focused nutrition education and screening for fatigue in primary care settings — may be warranted given our subgroup findings.

The Iranian dietary context differs substantially from Western populations studied previously; traditional Persian diets emphasize rice, legumes and minimally processed foods, but urbanization has increased UPF availability and consumption. Cultural factors (such as sodium intake patterns) and sex-specific nutritional deficiencies (e.g., zinc) may modulate the impact of UPFs and could partly explain why associations were stronger in men in our sample. These contextual considerations limit generalizability and underscore the need for region-specific research and tailored interventions.

## Conclusion

Our findings indicate that higher UPFs intake was significantly associated with the risk of frailty and exhaustion among community-dwelling older adults, especially for men. These findings underscore the potential impact of dietary quality on functional aging and support the hypothesis that UPFs may contribute to biological mechanisms underlying frailty (including systemic inflammation and adiposity). While causality cannot be inferred due to the cross-sectional design, the observed associations highlight UPF consumption as a modifiable risk factor in older populations.

These results reinforce the importance of promoting minimally processed, nutrient-dense dietary patterns as part of healthy aging strategies. By addressing a previously understudied area, our study contributes to the development of more targeted nutritional guidelines that reflect the specific needs and challenges of older adults. While our cross-sectional design prevents causal claims, these findings highlight UPF consumption as a plausible, modifiable risk factor worthy of further longitudinal and interventional study. Future prospective and mechanistic research including analyses of gut microbiota, neuroinflammation, food-matrix effects, and mediation by inflammation and adiposity is needed to confirm these associations and to inform tailored public-health strategies.

## Supplementary Information

Supplementary Material 1

## Data Availability

No datasets were generated or analysed during the current study.
