# Supplementary material for: Association between ultra-processed foods intake and frailty risk in community-dwelling older adults
Source: J Health Popul Nutr. 2025 Dec 25;45:30. doi: 10.1186/s41043-025-01209-2 (PMC12849376; doi:10.1186/s41043-025-01209-2)
Supplement: Supplementary file 1 — Supplementary Material 1 [file 41043_2025_1209_MOESM1_ESM.docx]

| **Supplementary Table 1:** List of items used for UPFs evaluation | |
| --- | --- |
| 1 | Industrial bread and packaged sandwich breads (highly processed) |
| 2 | French Fries |
| 3 | Industrial fruit drinks/fruit beverages |
| 4 | Packaged biscuits/cookies |
| 5 | Crackers |
| 6 | Jam |
| 7 | Homemade Cakes |
| 8 | Industrial snack cakes |
| 9 | Soybeans |
| 10 | Hamburgers |
| 11 | Sausages |
| 12 | Kalbas |
| 13 | Pizza |
| 14 | Sweetened condensed milk and flavored milk drinks |
| 15 | Packaged savory snacks (potato chips, crisps) |
| 16 | Processed cheese and cheese spreads |
| 17 | Traditional Ice Cream |
| 18 | Ice-cream and industrial frozen desserts |
| 19 | Margarine and industrial spreads |
| 20 | Red Sauce |
| 21 | Mayonnaise |
| 22 | Puff |
| 23 | Chocolate |
| 24 | Dry pastry |
| 25 | Fresh pastry |
| 26 | Gaz |
| 27 | Candy |
| 28 | Soft drink |
| 29 | Sohan |
| 30 | Creamy caramel |
| 31 | Sweet halva |
